# Happy or Silent Hypoxia in COVID-19–A Misnomer Born in the Pandemic Era

**DOI:** 10.3389/fphys.2021.745634

**Published:** 2021-10-18

**Authors:** Evangelia Akoumianaki, Katerina Vaporidi, Maria Bolaki, Dimitris Georgopoulos

**Affiliations:** Department of Intensive Care, School of Medicine, University Hospital of Heraklion, University of Crete, Heraklion, Greece

**Keywords:** peripheral chemoreceptors, dyspnea, metabolic hyperbola, ventilation curve, brain curve, respiratory system compliance

Early in the pandemic era of COVID-19 the term “happy or silent hypoxia” was introduced to describe patients with COVID-19 who presented with severe hypoxemia and absence of dyspnea (Couzin-Frankel, 2020; Guan et al., 2020). The absence of dyspnea despite severe hypoxemia was considered to be a “paradox” and unique to COVID-19 (Archer et al., 2020; Ferenchick and Ferenchick, 2020). As such the term “happy or silent hypoxia” has been received wide attention by the press and social media and even described as a silent killer in COVID-19 (Levitan, 2020). Although scientific evidence is lacking, central nervous system viral invasion has been put forward to explain this “paradox” (Nouri-Vaskeh et al., 2020; Gopal et al., 2021; Tavčar et al., 2021). Nevertheless, basic principles of respiratory system physiology dictate that the absence of dyspnea despite severe hypoxemia is not specifically linked to COVID-19 but to other lung diseases as well (Tobin et al., 2020). The aim of our article is to present information about the responses to both acute and sustained hypoxia and provide an analysis of control of breathing physiology that could explain the absence of dyspnea despite severe hypoxemia. Specifically, we apply in hypoxemic patients with COVID-19 our currently published analysis (Vaporidi et al., 2020) that relates arterial carbon dioxide levels with respiratory centers response to this stimulus, contrasting the brain's responses to the patient's ability to generate effective alveolar ventilation. This analysis may facilitate comprehension of the pathophysiology of dyspnea in hypoxemic patients with COVID-19.

## Hypoxemia and Dyspnea

Hypoxemia stimulate the carotid bodies, small clusters of oxygen pressure sensitive cells located at the carotid bifurcation, which via glossopharyngeal nerve increase the activity of the respiratory center in medulla oblongata (Vaporidi et al., 2020). The increased respiratory center output travels (inspiratory flow-generation pathway) from the brainstem and upper cervical spine neurons to the nucleus of respiratory motoneurons, leading to augmented activation and contraction of the inspiratory muscles and finally to an increase in inspiratory flow and thus, depending on the respiratory rate, ventilation (Vaporidi et al., 2020). The augmented respiratory center activity is simultaneously transmitted up to the cerebral cortex (corollary discharge) and produces the subjective unpleasant symptom of dyspnea, independently of the type of primary stimulus (Moosavi et al., 2003). It follows that dyspnea is caused by the cerebral cortex projection of respiratory center activity.

At first glance, minimal or even absence of dyspnea on a background of severe hypoxemia appears “paradoxical.” Notwithstanding that dyspnea is a subjective symptom, understanding of the control of breathing mechanisms may explain this “paradox.”

## Hypoxemia and Respiratory Centers Activity

Since the quantification of the cortical projection of respiratory centers activity is not possible, ventilation and indices of respiratory effort per breath are used to estimate the respiratory center activity during hypoxemia. Acute progressive isocapnic hypoxemia increases ventilation in a hyperbolic manner; ventilation remains almost unchanged as PaO_2_ drops to ~60 mmHg, but at lower PaO_2_, it increases progressively with hypoxemia (Weil et al., 1970). The increase in ventilation is mainly due to an increase in tidal volume (i.e., change in effort per breath, respiratory drive) and not in respiratory rate (Vaporidi et al., 2020). Although PaO_2_ and not SaO_2_ is the stimulus to carotid bodies, to overcome the difficulties of the non-linear relationship between PaO_2_ and ventilation, the sensitivity to hypoxia is usually expressed by the linear relationship between ventilation or mouth occlusion pressure (P_0.1_) and SaO_2_. Though a wide range of normal hypoxic ventilatory response is observed, progressive isocapnic hypoxia in normal young adults increases ventilation on average by 0.8 l/min/%SaO_2_ and P0.1 by 0.2 cm H_2_O/%SaO_2_ (Peterson et al., 1981). Thus, a progressive drop of SaO_2_ from 97 to 80% (PaO_2_≈45 mmHg) increases ventilation, mainly due to tidal volume increase, by ≈14l/min and P0.1 by ≈5 cm H_2_O. Corne et al. showed that an acute decrease of SaO_2_ to 80% increases peak inspiratory muscle pressure by an average of 8 cm H_2_O, a value that represents only 5–8% of maximum inspiratory pressure (Corne et al., 2003). This increase in effort per breath, due to corresponding increase in output from respiratory centers, may not be associated with dyspnea. Moosavi et al. showed that at PaO_2_ 40–45 mmHg air hunger (equivalent to dyspnea) with free unconstrained breathing virtually did not exist, averaging <15% in visual analog scale (VAS) (Moosavi et al., 2003). Even with constant ventilation, constrained to resting level, strong air hunger (>40% in VAS) at this level of hypoxemia was not present in one-half of the subjects. The relatively low response to hypoxia is considerably attenuated at hypocapnic levels (PaCO_2_ <39 mmHg) and virtually lost when PaCO_2_ is reduced by ~10 mm Hg relative to eupnea (Corne et al., 2003). Furthermore, compared to young adults, in elderly normal subjects the response to acute hypoxemia is reduced by ~50% (Peterson et al., 1981), while in patients with type II diabetes is virtually blunted (Nishimura et al., 1989; Weisbrod et al., 2005).

**Figure 1:**
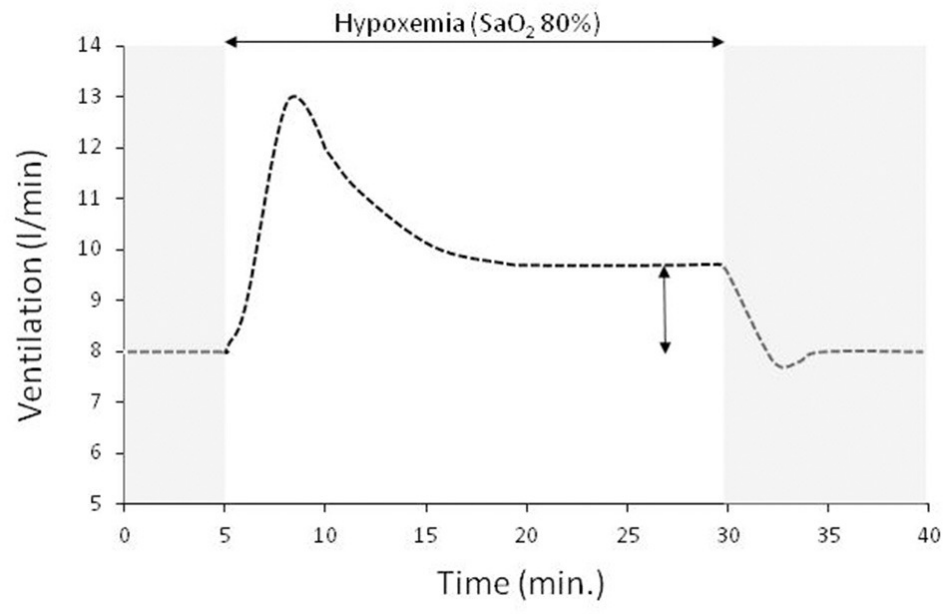
A typical ventilatory response to sustained isocapnic hypoxemia (horizontal double edge arrow) preceded and followed by room air breathing (gray area, SaO_2_ 97%). In this experiment the drop of SaO_2_ to 80% is achieved within 2 min and remained at this level for 25 min. Sustained isocapnic hypoxemia results in an initial brisk increase in ventilation which then declines to a plateau that is ~20% higher than that at room air breathing (vertical double edge arrow).

Sustained hypoxemia (i.e. lasting >15 min.) is more relevant to disease than acute brief hypoxemia. In adult humans the ventilatory response to acute sustained hypoxia (SaO_2_ 80%) is biphasic, characterized by an initial brisk increase followed by a decline to a plateau, slightly higher than that during normoxia (Figure 1) (Easton et al., 1986). This inhibitory effect of hypoxemia on ventilation is independent of the CO_2_ stimulus (Georgopoulos et al., 1989a) and can persist for several days in humans during sustained hypoxia at high altitude (Sato et al., 1994; Hupperets et al., 2004). However, despite this inhibition the ventilator pump can respond promptly to CO_2_ (Georgopoulos et al., 1990). Compared to the initial increase in ventilation, the reduction of hypoxic ventilatory response is of central origin since carotid sinus nerve activity remains unchanged during hypoxemia and ventilation followed the decrease in phrenic nerve activity (Vizek et al., 1987). The most likely mechanism of this inhibitory effect of sustained hypoxemia is the central modulation of carotid bodies afferents, since the magnitude of hypoxic ventilatory decline is proportional to the initial increase and its expression necessitates the presence of carotid bodies (Georgopoulos et al., 1989b; Long et al., 1993). This depressant effect of hypoxemia indicates that the relatively low increase in respiratory center activity during acute hypoxemia will be further decreased if the hypoxic stimulus is sustained. Indeed, it has been shown that during sustained hypoxia with free unrestrained breathing or constrained constant ventilation air hunger mirrors the biphasic ventilatory response (Chonan et al., 1998; Moosavi et al., 2004). Furthermore, compared to constrained constant ventilation, air hunger is considerably reduced by free unrestrained breathing (Moosavi et al., 2004), indicating that satisfying hypoxic ventilatory demands relieve dyspnea; with free unrestrained breathing during sustained hypoxemia air hunger at plateau ventilation averaged <10% in visual analog scale, a value that is not even noticeable by most subjects.

Although hypoxemia *per se* is a weak stimulus of respiratory centers it may increase their activity indirectly by increasing the ventilatory response to CO_2_ (Mohan and Duffin, 1997). Therefore, during hypoxemia respiratory center activity is increased to lower resting PaCO_2_ and at the same time low PaCO_2_ decreases or even abolishes hypoxic output of respiratory centers (Corne et al., 2003).

## Hypoxemia and Dyspnea In Acute Lung Disease

**Figure 2:**
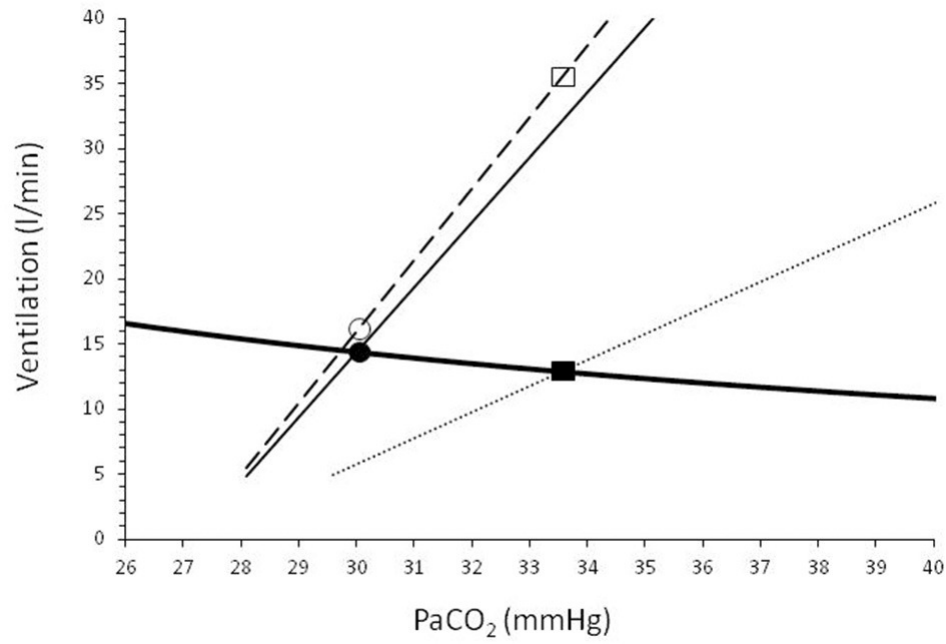
Graphical representation of the metabolic hyperbola (solid heavy line) and brain/ventilation curve of a patient with COVID-19 pneumonia. CO_2_ production and dead space to tidal volume ratio were considered higher than normal (V'CO_2_: 250 ml/min, V_D_/V_T_: 0.5). Dashed line shows brain curve. Ventilation curves at two values of respiratory system compliance, are shown by solid (slightly reduced compliance) and dotted (low compliance) lines. The intersection of the metabolic hyperbola and ventilation curve determines the steady-state PaCO_2_. Early in the disease, where phenotype 1 may prevail (slightly reduced compliance), the deviation between brain and ventilation curve is small and, as a result, the actual PaCO_2_ is slightly higher than the desired PaCO_2_ (30.0 vs. 29.6 mmHg). The increased activity of respiratory center as a result of this small difference between actual and desired PaCO_2_ increases the ventilatory demands to 16.0 l/min (open circle), only 1.2 l/min higher than the actual minute ventilation (V'_E_, 14.8 l/min, closed circle). Later in the disease compliance decreases (i.e., type 2 phenotype prevails), causing a greater deviation between brain and ventilation curve. PaCO_2_ increases to 33.5 mmHg and actual V'_E_ decreases to 12.8 l/min (closed rectangular). At this level of PaCO_2_ the brain curve dictates (open rectangular) that respiratory center activity corresponds to V'_E_ of 35.2 l/min. This high respiratory center activity combined with unmet ventilatory demands (35.2–12.8 = 22.4 l/min) causes severe dyspnea and respiratory distress.

In patients with acute lung disease such as in patients with acute respiratory distress syndrome (ARDS) due to COVID-19, the respiratory center activity is often elevated due to increased sensitivity to CO_2_ for several reasons (i.e., metabolic acidosis, hypoxemia, neurotransmitters affecting the brain stem, stimulation of lung and chest wall receptors) (Vaporidi et al., 2020) and result in hypocapnia, which sometimes is significant (Wang et al., 2020). If the inspiratory flow generation pathway and particularly respiratory system compliance is near-normal, the increased inspiratory motor output results in a tidal volume that is only slightly lower than that desired by the respiratory center (Vaporidi et al., 2020). It is well-known that some patients with COVID-19 pneumonia who meet criteria for ARDS, have relatively preserved respiratory system compliance and vasculopathy is the main cause of hypoxemia (Gattinoni et al., 2020). Under these circumstances, dyspnea and respiratory distress may be minimal because (1) hypoxemia, particularly in elderly patients and with co-morbidities such as type 2 diabetes, is a weak stimulus of respiratory center, (2) low PaCO_2_ (due to hypoxemia, and other causes related to critical illness) further decreases the hypoxic drive, and (3) PaCO_2_ desired by the respiratory center is similar to or slightly lower than the actual PaCO_2_ (Figure 2). The desired PaCO_2_ is a theoretical value defined by the intersection point between the “brain curve” and metabolic hyperbola (the graphical representation of the alveolar air equation for CO_2_) (Vaporidi et al., 2020). The term “brain curve” represents the ventilation desired by the brain at any PaCO_2_ level (Vaporidi et al., 2020). The actual PaCO_2_ is determined by the intersection point between “ventilation curve” and metabolic hyperbola. The term “ventilation curve” describes the actual changes in minute ventilation in response to changes in PaCO_2_, as modified by any impairment in respiratory system mechanics and respiratory muscle function (Vaporidi et al., 2020). Patients with COVID-19 pneumonia, at least initially, have normal respiratory muscle function and, some of them, slightly reduced respiratory system compliance (Gattinoni et al., 2020). In these patients the ventilation curve deviates slightly from the brain curve (because of a mild decrease in compliance), causing a minimal increase of the actual PaCO_2_ (Figure 2). The mild increase in respiratory center activity, due to higher than desired PaCO_2_, is met by a corresponding increase in inspiratory motor output per breath (Akoumianaki et al., 2019; Vaporidi et al., 2020), which may not be sufficiently high to result in dyspnea and distress. With disease progression, similar to classical ARDS, deterioration of respiratory system mechanics occurs, causing a greater deviation of the ventilation from the brain curve and dyspnea and respiratory distress may ensue (Figure 2), mainly due to unmet ventilatory demands (Mendonca et al., 2014).

Finally, we must realize that carotid bodies respond to PaO_2_ and not to SaO_2_. The relationship between PaO_2_ and SaO_2_ is described by a sigmoid shape curve, the oxygen dissociation curve, which is shifted to the right (for a given PaO_2_ SaO_2_ decreases) by high temperature. High fever, common in COVID-19 and other acute lung diseases, including classical ARDS, can cause substantial desaturations without any change in PaO_2_ and thus in peripheral chemoreceptor stimulation of respiratory centers. It follows that, compared to PaO_2_, SaO_2_ overestimates the degree of hypoxemia at the presence of high fever.

In conclusion, patients with COVID-19 pneumonia may present without dyspnea, despite severe hypoxemia. The absence of dyspnea is not specifically related to COVID-19 but may occur in any patient with acute hypoxemic respiratory failure exhibiting normal respiratory muscle function and relatively normal respiratory system mechanics.

## Author Contributions

DG conceived the idea. DG, EA, KV, and MB performed the literature search and drafted the manuscript. The article was critically reviewed and revised by all authors. All authors read and approved the final manuscript.

## Funding

Special Account for Research Funds University of Crete.

## Conflict of Interest

The authors declare that the research was conducted in the absence of any commercial or financial relationships that could be construed as a potential conflict of interest.

## Publisher's Note

All claims expressed in this article are solely those of the authors and do not necessarily represent those of their affiliated organizations, or those of the publisher, the editors and the reviewers. Any product that may be evaluated in this article, or claim that may be made by its manufacturer, is not guaranteed or endorsed by the publisher.
